# Lipidic cubic phase injector is a viable crystal delivery system for time-resolved serial crystallography

**DOI:** 10.1038/ncomms12314

**Published:** 2016-08-22

**Authors:** Przemyslaw Nogly, Valerie Panneels, Garrett Nelson, Cornelius Gati, Tetsunari Kimura, Christopher Milne, Despina Milathianaki, Minoru Kubo, Wenting Wu, Chelsie Conrad, Jesse Coe, Richard Bean, Yun Zhao, Petra Båth, Robert Dods, Rajiv Harimoorthy, Kenneth R. Beyerlein, Jan Rheinberger, Daniel James, Daniel DePonte, Chufeng Li, Leonardo Sala, Garth J. Williams, Mark S. Hunter, Jason E. Koglin, Peter Berntsen, Eriko Nango, So Iwata, Henry N. Chapman, Petra Fromme, Matthias Frank, Rafael Abela, Sébastien Boutet, Anton Barty, Thomas A. White, Uwe Weierstall, John Spence, Richard Neutze, Gebhard Schertler, Jörg Standfuss

**Affiliations:** 1 Laboratory for Biomolecular Research, Paul Scherrer Institute, Villigen 5232, Switzerland; 2 Department of Physics, Arizona State University, Tempe, Arizona 85287, USA; 3 Center for Free-Electron Laser Science, Deutsches Elektronen-Synchrotron DESY, 22607 Hamburg, Germany; 4 Biometal Science Laboratory, RIKEN SPring-8 Center, Hyogo 679-5148, Japan; 5 SwissFEL, Paul Scherrer Institute, Villigen 5232, Switzerland; 6 Linac Coherent Light Source (LCLS), SLAC National Accelerator Laboratory, Menlo Park, California 94025, USA; 7 PRESTO, JST, Saitama 332-0012, Japan; 8 Department of Chemistry and Biochemistry, and Center for Applied Structural Discovery, Biodesign Institute, Arizona State University, Tempe, Arizona 85287-1604, USA; 9 Department of Chemistry and Molecular Biology, University of Gothenburg, Box 462, SE-40530 Gothenburg, Sweden; 10 SACLA Science Research Group, RIKEN/SPring-8 Center, Hyogo 679-5148, Japan; 11 Department of Cell Biology, Kyoto University, Kyoto 606-8501, Japan; 12 Department of Physics, University of Hamburg, 22761 Hamburg, Germany; 13 Centre for Ultrafast Imaging, University of Hamburg, 22761 Hamburg, Germany; 14 Lawrence Livermore National Laboratory, Livermore 94550, USA; 15 Department of Biology, ETH Zurich, Zürich 8093, Switzerland

## Abstract

Serial femtosecond crystallography (SFX) using X-ray free-electron laser sources is an emerging method with considerable potential for time-resolved pump-probe experiments. Here we present a lipidic cubic phase SFX structure of the light-driven proton pump bacteriorhodopsin (bR) to 2.3 Å resolution and a method to investigate protein dynamics with modest sample requirement. Time-resolved SFX (TR-SFX) with a pump-probe delay of 1 ms yields difference Fourier maps compatible with the dark to M state transition of bR. Importantly, the method is very sample efficient and reduces sample consumption to about 1 mg per collected time point. Accumulation of M intermediate within the crystal lattice is confirmed by time-resolved visible absorption spectroscopy. This study provides an important step towards characterizing the complete photocycle dynamics of retinal proteins and demonstrates the feasibility of a sample efficient viscous medium jet for TR-SFX.

## Abstract (web summary)

Serial femtosecond crystallography using X-ray free-electron lasers has huge potential for time-resolved structural experiments. Here, the authors present a structure of the light-driven proton pump bacteriorhodopsin using these techniques.

## Graphical abstract

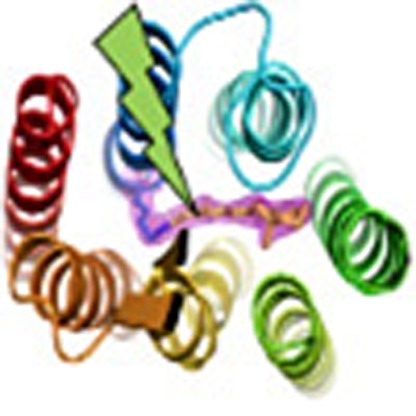

Serial femtosecond crystallography (SFX) is an emerging method for the structure determination of proteins using X-ray free-electron lasers (XFELs)^1^,^2^. In SFX, a continuous supply of crystals is delivered into a pulsed XFEL beam. If a crystal is hit by the high-intensity XFEL pulse, it will be destroyed but deliver a ‘snapshot' diffraction pattern according to the ‘diffraction before destruction' principle^3^. By merging thousands of such ‘snapshot' diffraction patterns a complete data set can be obtained. A technical challenge in SFX is the need to maintain a constant stream of fresh sample during data collection. In early SFX studies of soluble^4^ and membrane proteins^5^,^6^, a liquid jet injector was used to deliver crystals^7^, requiring up to grams of well-diffracting microcrystals. Such quantities are difficult to obtain for most proteins and are a particular challenge for membrane proteins that are expressed recombinantly. Other injectors, such as elektrokinetic^8^,^9^,^10^ and pulsed liquid droplet^11^ systems, have been developed to reduce sample consumption. To date, the most successful is an injector that extrudes crystals in a highly viscous lipidic cubic phase (LCP)^12^. Because of its high viscosity, LCP can be extruded very slowly (typically 0.05 to 2 μl per minute) allowing for a very sample efficient data collection (∼100–500 μg of protein per 10,000 indexed diffraction patterns). Another advantage is that such lipidic mesophases have been successfully used for crystallization of membrane proteins and in particular pharmacologically relevant G-protein-coupled receptors. Because of these advantages several new membrane protein structures have been solved using LCP-SFX^13^,^14^,^15^,^16^,^17^. Alternative viscous media have been shown to be a promising option for soluble proteins^18^,^19^,^20^. While still a very young technique, the development of LCP-SFX has already led to the first structures of a membrane-bound protein complex^21^, and of membrane proteins with increased structural resolution^13^ and native-like dynamics^14^.

A key advantage of using ultrafast XFEL pulses in structural biology, is the possibility for time-resolved pump-probe measurements (TR-SFX) in a wide temporal range from femtoseconds to milliseconds at physiological temperatures. The potential of XFELs for such time-resolved crystallographic studies at near atomic resolution has recently been demonstrated using photoactive yellow protein^22^ and myoglobin^23^. These studies made use of a sample-expensive liquid jet to deliver crystals and an optical pump laser synchronized with the XFEL to obtain X-ray diffraction snapshots from photoactivated protein states. An increasing time delay between the pump laser and the probing XFEL pulse allows for the recording of sequential stages of the activation mechanism. While each shot at any given time delay provides a single snapshot from microcrystals in a random orientation, many shots together provide enough data for a reconstruction of three-dimensional electron density maps. By collecting different time delays, it is possible to assemble molecular movies of light-induced conformational changes in proteins. Since LCP injectors are very efficient in sample usage, they could open up TR-SFX for a large range of proteins that cannot be produced in large quantities.

A further advantage of XFEL-based SFX over conventional diffraction strategies stems from the diffraction before destruction characteristics of the XFEL pulses; they contain ∼10^12^ X-ray photons in a femtoseconds time range and this high fluence is sufficient to produce high-resolution diffraction patterns, while at the same time outrunning most radiation damage processes^3^,^24^. Radiation damage otherwise often obscures biological interpretation, especially in proteins containing radiation sensitive co-factors such as metal–ion clusters^25^,^26^,^27^, making it a fundamental barrier that limits correct structure determination. Moreover, in the ultrafast regime, conformational changes tend to be small and are thus easily obscured by the radiation damage. The unique abilities of XFEL pulses to outrun the structural radiation damage and to measure very fast processes are complementary.

Proteins that bind the common biological chromophore retinal are ideal for time-resolved measurements, since they can be activated with a flash of laser light^28^. These proteins fulfil important biological functions, for example, in visual signalling as G-protein-coupled receptors^29^. Using an approach known as optogenetics, retinal-binding light-gated ion channels are employed to light-activated specific neurons with short light pulses. The classical model to study retinal proteins is the light-driven proton pump bacteriorhodopsin (bR). Here we present the crystal structure of bR solved by SFX to 2.3 Å resolution using an XFEL. Difference Fourier maps obtained from a pump-probe experiment with a time delay of 1 ms are compatible with the dark to M state transition and demonstrate the possibility to use an LCP injector for time-resolved crystallography. In addition, we demonstrate that TR-SFX experiments synergize well with serial millisecond crystallography (SMX) at a synchrotron microfocus beamline^30^ for pretesting crystalline sample and benefit from timing optimization, using *in situ* time-resolved absorption spectroscopy. This study represents an important step towards fulfilling the promise of XFEL technology to advance structural biology from the determination of molecular snapshots to molecular movies.

## Results

### Experimental set-up

In contrast to crystals obtained in a liquid solution, crystals grown in LCP can only be diluted not concentrated. To nevertheless allow for a high hit rate (percentage of images with diffraction patterns) and efficient data collection, we adjusted crystallization conditions based on our experience with SMX at synchrotron sources^30^. By increasing the precipitant and protein concentrations during crystallization to 38% and 15 mg ml^−1^, we increased nucleation and the number of small 10–15 μm sized crystals to achieve hit rates of 5–10% at the XFEL. Importantly, smaller crystals facilitate time-resolved measurements by allowing the pump laser to fully penetrate the crystal and maximize activation of proteins within (compare section light-induced conformational changes). This is particularly important when using femtosecond laser activation, since it is well known that high photo-product yields are more difficult to achieve using very short photoexcitation pulses. Data collection was performed at the Coherent X-ray Imaging (CXI) endstation of the Linac Coherent Light Source (LCLS), SLAC National Accelerator Laboratory, using XFEL pulses of 75 fs duration and focused to a full-width at half-maximum of 1.5 × 1.5 μm^2^. The average energy of an XFEL pulse was 3.1 mJ, but the pulses were attenuated to ∼2% of their full intensity to protect the Cornell-SLAC Pixel array detector from being damaged by intense Bragg peaks. X-ray pulses were delivered at 120 Hz repetition rate, while a femtosecond optical laser provided pumping light pulses at a rate of 30 Hz with an initial pump-probe delay of 1 ms. Diffraction patterns were collected in a single 12 h shift. Overall the quality of the collected data is comparable to the data we collected using SMX at a synchrotron (Table 1).

### Comparison of SFX with SMX and Cryo structures

We solved the SFX dark-state structure of bR using sensory rhodopsin II (PDB: 1H68 (refs 31, 32, 33, 34)) for molecular replacement. The following refinement procedure was identical to our previous strategy of processing SMX data collected at a synchrotron microfocus beamline^30^. This allowed for an easy comparison between the two studies. In both cases, we use an automatic model building with simulated annealing to minimize the effect of model bias.

**Figure 1:**
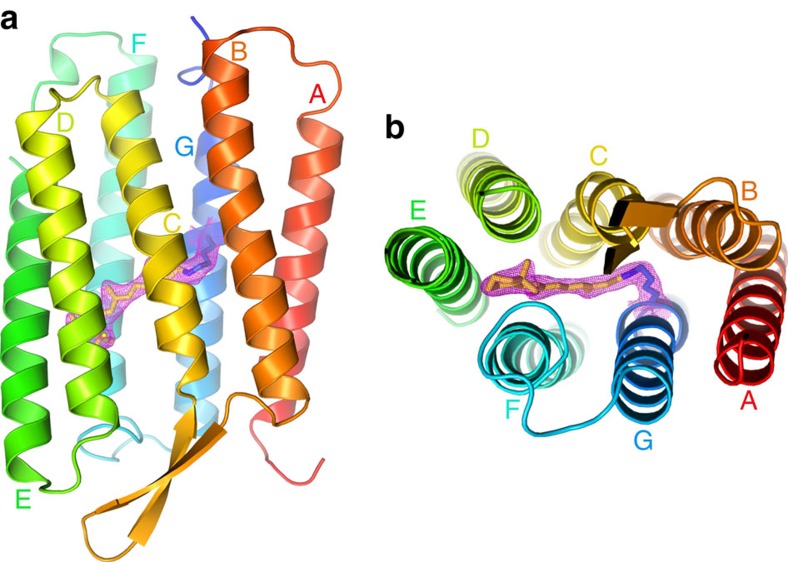
Structure of bR in dark-state determined by SFX. In side view (**a**) and perpendicular to the membrane (**b**). The retinal is shown as yellow sticks and the Lys216 side chain covalently binding retinal as blue sticks. Continuous 2*F*_o_−*F*_c_ electron density map around retinal and lysine is shown in magenta. Helices A–G are defined based on the header of pdb entry 1QHJ (TM A: 6–32; TM B: 37–58; TM C: 80–100; TM D: 105–127; TM E: 131–160; TM F:165–191; and TM G: 201–224).

**Figure 2:**
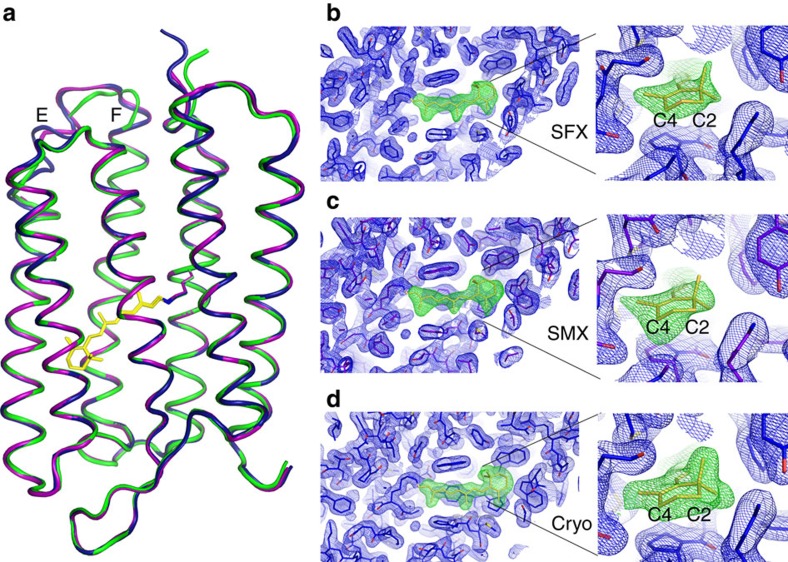
Comparison of bR dark-state structures. Overall structures determined by SFX (purple), SMX (blue) and conventional cryo-crystallography (green). (**a**) Overall structures are highly similar with only minor deviations in loop regions and the termini. (**b**–**d**) Comparison of electron density maps in the retinal-binding pocket (SFX (**b**), SMX (**c**) and Cryo (**d**)). The electron density maps (blue, 2*F*_o_−*F*_c_, 1 *σ*) are well defined in all three cases and strong positive density is observed when retinal (yellow sticks; left panel) is omitted during refinement (green, *F*_o_−*F*_c_, 2.5 *σ*). Flexible regions of the retinal β-ionone ring (insets), which are not part of the retinal conjugated double bond system, can be clearly identified in the two room temperature structures.

The SFX structure of bR (Fig. 1) contains a single protein molecule in the asymmetric unit with a covalently bound retinal chromophore, 17 water molecules and 5 lipid fragments. The protein backbone is well resolved except for the residues 1–4 (N terminus), 155–157 (loop) and 232–249 (C terminus). The obtained electron density of the retinal-binding pocket is shown in the Supplementary Fig. 1. The overall structure is similar to previously reported bR structures and to our structures determined by SMX and cryo-crystallography (Cryo). The root mean square deviation (r.m.s.d.) for C_α_ atoms with our earlier cryo-structure (PDB: 4X32) is 0.50 Å^2^, while the r.m.s.d. to our room temperature SMX structure (PDB: 4X31) is, at 0.29 Å^2^, slightly smaller. The largest difference in the protein backbone trace between the structure determined under cryogenic conditions and the two room temperature structures is observed in the cytoplasmic loop between helices E and F (Fig. 2). Note that not all residues in this region could be unequivocally assigned to electron density, indicating an overall high flexibility in this region (Supplementary Fig. 2).

In the interior of the protein around the retinal chromophore, the three data collection strategies (SFX, SMX and Cryo) lead to 2*F*_o_−*F*_c_ electron density maps of comparatively high quality (Fig. 2). Omitting the retinal before calculation of the maps resulted in clear positive *F*_o_−*F*_c_ difference electron density in all the three cases. Interestingly, the OMIT electron density map around the C2–C4 atoms of the retinal β-ionone ring is partially depleted in case of the SMX and SFX structures. This is the only part of the retinal molecule that is not constrained by delocalized *π*-electrons and thus retains some structural flexibility. It is likely that this region of the retinal exists in multiple conformers at ambient temperatures, which are then sampled by both of the room temperature serial crystallographic experiments. The cryogenic experiment, on the other hand, resulted in a well-defined electron density, probably because this conformer is thermodynamically favoured at the non-physiological temperature.

### Radiation damage

Radiation damage is one of the primary limiting factors in obtaining structural information from weakly diffracting membrane protein crystals. The problem is further intensified when very small microcrystals are needed for efficient photoactivation in a pump-probe experiment. Often, radiation damage is avoided by collecting small portions of data from a larger number of crystals, for example, by using raster scanning in cryo-loops^32^,^34^ or other fixed-target mounting devices^35^. Injector-based serial crystallography pushes the principle to the extreme by exposing each crystal to X-rays only once, while providing a continuously refreshed supply of thousands of unexposed crystals. In contrast to the ∼0.7 MGy deposited per bR crystal in our previous synchrotron experiment^30^, each free-electron laser X-ray pulse subjected a crystal to a dose of 10 MGy, far above the suggested limit of under 1 MGy at room temperature^36^,^37^. Nevertheless, at the used pulse energy (∼62 μJ after attenuation) and pulse duration of 75 fs (∼6.57e^10^ photons per pulse), the global radiation damage is not to be expected based on studies with the model system lysozyme^4^,^38^.

**Figure 3:**
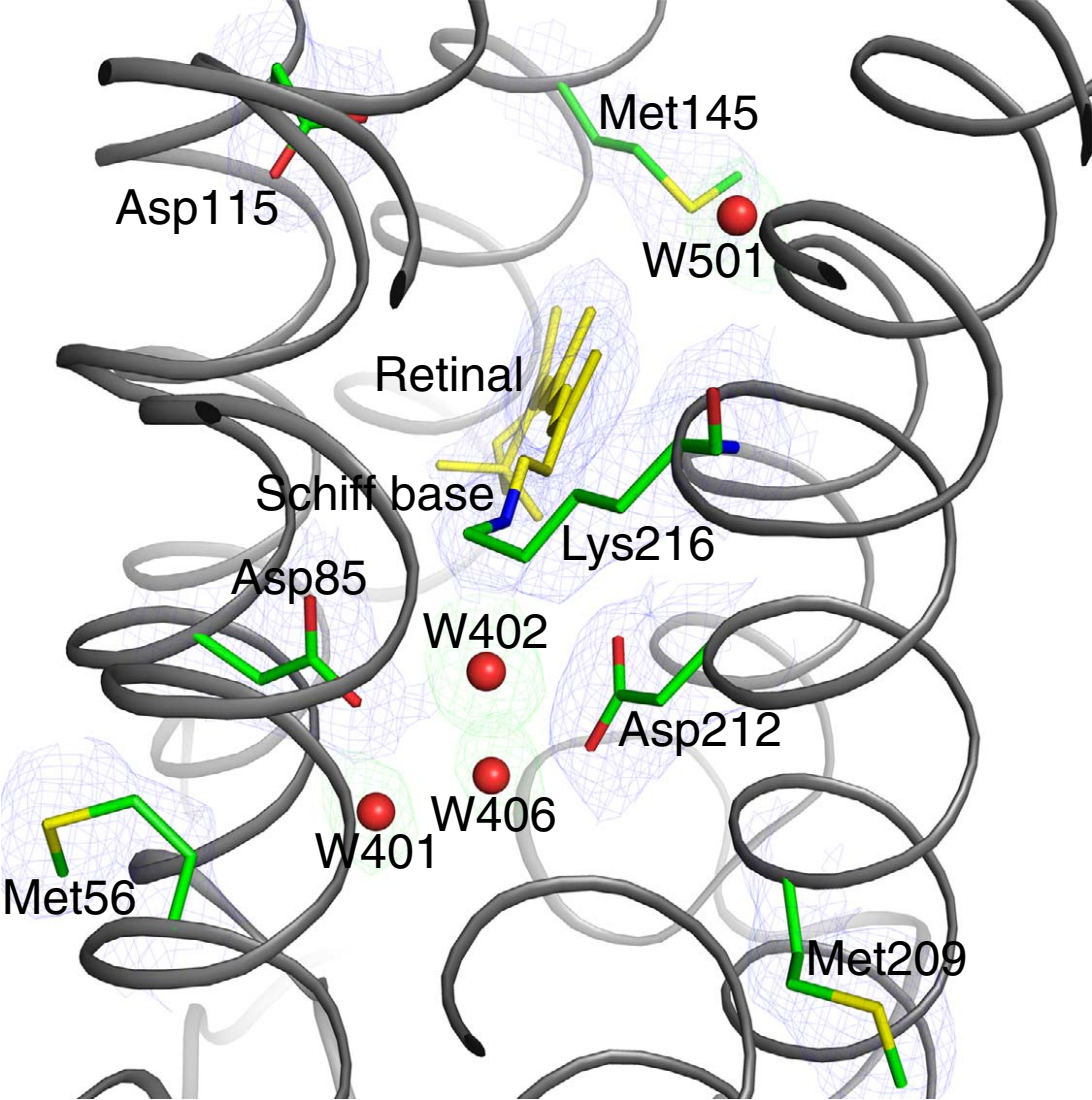
Radiation sensitive residues around the retinal Schiff base link. The region is critical because proton pumping by bR requires light-driven *trans*–*cis* isomerization of retinal, rearrangement of water molecules (W401, W402 and W406) and changes of Asp85 and Asp212. Refinement of our SFX data (bR_dark_) results in well-resolved electron density (purple, 2*F*_o_−*F*_c_, 1 *σ*) for amino-acid side chains involved in this mechanism. Omission of water molecules results into strong positive difference peaks (green, *F*_o_−*F*_c_, 2.5 *σ*) indicating well-ordered structural water molecules.

Decarboxylation of Asp and Glu residues occurs as one of the earliest signs of radiation damage in protein crystallography, long before the global radiation damage manifests itself through the loss of diffraction power^39^. An electron density depleted region in the expected position of the residue side chain can therefore be used as an early indicator of the radiation damage. These signs compatible with the initial radiation damage can be found for example in Asp38, Asp102 and Asp104 in the structure collected at cryogenic temperatures at the synchrotron, while those residues are well resolved in the two structures from serial crystallography (Supplementary Fig. 3). Asp and Glu residues are crucial for the proton-exchange mechanisms necessary for bR to function as a proton pump (for a review see ref. 40). In particular, the cluster formed by Asp82, Asp85 and three water molecules (W401, W402 and W406) are crucial as the primary proton acceptor, ∼40 μs after the light-driven retinal isomerization from all-*trans* to 13-*cis* retinal. Light-induced conformational changes in this region, including rearrangements of water molecules are observed in cryo-trapped early photoactivation intermediates, but are also very similar to the changes induced by the X-ray radiation^41^. With continuous 2*F*_o_−*F*_c_ density for Asp82 and Asp85 and clear *F*_o_−*F*_c_ difference density for the three water molecules, our SFX structure of bR resolves this region well (Fig. 3). Furthermore, the SFX structure is very similar to the bR structure collected with low doses of synchrotron radiation under cryo-conditions (PDB: 4MD2)^42^, showing a local r.m.s.d. of 0.565 Å (117 atoms 8 Å around W402). These observations are consistent with the ability of femtosecond X-ray pulses to outrun the radiation damage, which is a prerequisite for resolving small conformational changes in a TR-SFX experiment.

### Light-induced conformational changes

**Figure 4:**
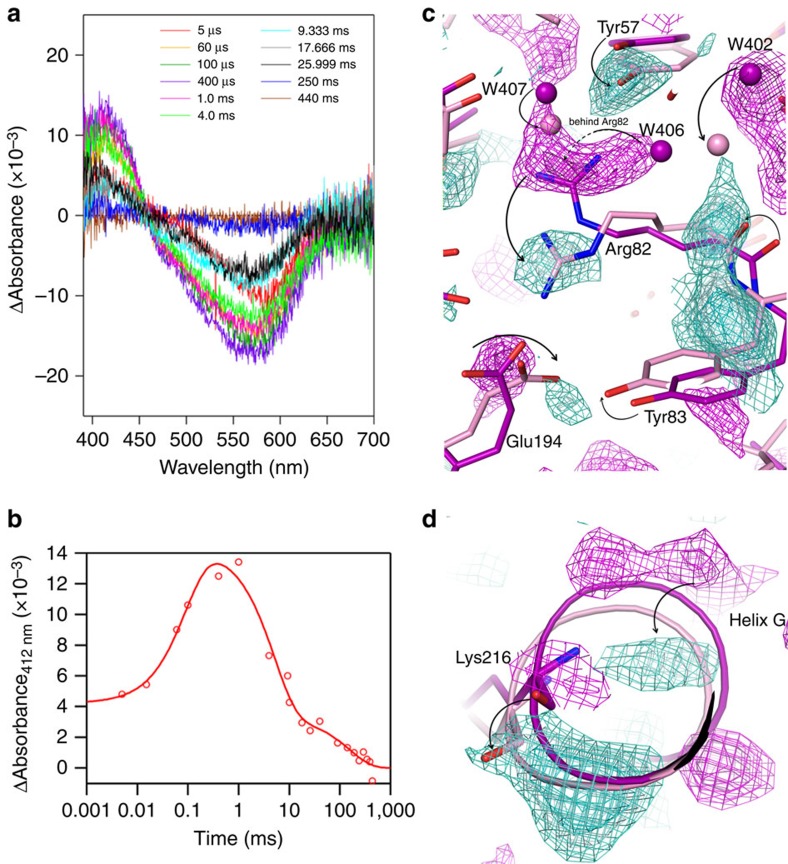
Time-resolved absorption spectroscopy and crystallography on bR crystals. Time-resolved absorption spectroscopy on bR crystals (**a**) indicates similar photocycle kinetics as observed in purple membranes^44^,^58^. Absorption changes at 412 nm (**b**) indicate maximum accumulation of the M intermediate at ∼1 ms after photoexcitation. The TR-SFX Fourier difference map with 1 ms time delay (*F*obs(bR1ms)-*F*obs(dark_bR); turquoise positive, magenta negative, shades represent 2.0, 2.5 and 3.0 *σ*) is compatible with known light-induced conformational changes characteristic for the bR M intermediate (dark (purple), M state (pink) and 1CWQ^49^). Reorganization in the proton transfer chain in the M intermediate involves a rotamer change of Arg82 and relocation of water molecules (**c**). Difference peaks around the site of retinal attachment Lys216 show a shift of helix G (**d**). Arrows indicate conformational changes apparent in the difference Fourier analysis.

An important consideration for time-resolved crystallography is whether the protein can be activated, while embedded within a crystal lattice. Furthermore, a TR-SFX experiment can be planned more efficiently if it is known at what time point after photoactivation conformational changes can be expected. We have employed time-resolved visible absorption spectroscopy to investigate bR activation within a crystal at room temperature and within the same LCP, as used for the TR-SFX experiment (Fig. 4). Light-minus-dark difference spectra obtained with this method are dominated by two major components: a decrease in absorption at 570 nm and an increase at 412 nm (Fig. 4a). The minima at 570 nm indicate a mixture of intermediate states that cannot be deconvoluted at this spectroscopic resolution. Absorption changes at 412 nm more specifically indicate the accumulation of the M intermediate of bR. The maximal amplitude of the 412 nm peak occurs ∼1 ms after photoactivation (Fig. 4b), which is in good agreement with the characterization of the bR photocycle in purple membranes using resonance Raman^43^, absorption^44^ and infrared^45^ spectroscopy. On the basis of these results, we performed TR-SFX using a 1 ms time delay. To exclude photo damage and formation of higher electronically excited levels by multi photon excitation^46^, we limited the laser power to 20 μJ. At sample position, this corresponds to 1–20 photons per retinal chromophore depending on the size of the crystal, its orientation with respect to the pump laser and scattering within the LCP.

The quantum efficiency of retinal activation (∼60% (ref. 47)) and the optical density of the crystals (∼2 OD in the long dimension) prevented full activation of all bR molecules within the crystal. Gradual subtraction of dark-state contribution in dark–state-subtracted electron density maps (*F*obs(bR1ms)−m*F*obs(dark_bR); Methods) indicates an active-state contribution of ∼13% (Supplementary Fig. 4; Supplementary Movie 1; dark–state-subtracted map^48^). This value is well in agreement with the occupancy of 11–13%, we obtained from occupancy refinement of the M state (PDB code: 1CWQ chain B) and our dark state against the bR_1 ms_ data. We further interpreted the X-ray diffraction data from structural intermediates by difference Fourier analysis, which represents the experimental changes in the X-ray diffraction data in real space using the resting state structural model for calculation of phases^31^. An important aspect of this analysis is that the resulting difference electron density has no phase bias towards the intermediate-state model, since that model never enters the calculation. The Fourier difference map calculated between dark data and bR_1 ms_ reveals a series of positive and negative difference peaks visible up to 5 *σ*. Strong difference density peaks ∼3 *σ* are only visible close to or within the protein molecules, but not in the surrounding or interior of the bR trimer (Supplementary Fig. 5). An absence of the difference peaks in the solvent channels where the light-induced changes are not expected indicates that the observed peaks within the molecules reflect differences between the bR_1 ms_ and dark-state structures. When superimposed on known structures of the bR resting and M states trapped at low temperature after thawing during illumination (PDB code: 1CWQ; chain A—dark state, 35% occupancy: resting state; chain B, 65% occupancy: M state)^49^, the Fourier difference maps highlight characteristic conformational changes upon photoactivation. For example, the protein backbone of helix G is clearly shifted around Lys216, the site of covalent retinal attachment (Fig. 4d). In another functionally important region, the Fourier difference maps indicate movement of the Arg82 backbone and a rotamer change of its side chain, both of that are associated with the M intermediate (Fig. 4c). We therefore infer that the difference Fourier maps reflect specific conformational changes in the bR photocycle.

## Discussion

New technological developments have had a decisive impact on the recent advances in the field of structural biology. Techniques originally developed for the crystallization^50^ and structure determination^51^ of bR, that is, crystallization in LCP and other lipidic phases, have become a routine method for the production of high-quality crystals of challenging human membrane proteins^52^. Serial injection technologies for crystals grown in LCP into the path of X-ray pulses from an XFEL have become a successful method for structure determination of novel membrane receptors^13^ and signalling complexes^21^. A main limiting factor in serial crystallography has always been the scarce resource of XFEL beamtime. However, crystals can be tested by serial injection at the synchrotron and the resulting data used to optimize experimental conditions for a time-resolved experiment at an XFEL. All sites currently building or already operating XFELs also run synchrotron beamlines for X-ray crystallography. Our studies thus provide an example of how synergy between these facilities can be used to allocate scarce beamtime and improve efficiency in structural biology.

Serial crystallography at XFELs overcomes limitations of classical crystallography by allowing us to collect data from small micrometre-sized crystals and to outrun radiation damage using intense femtosecond X-ray pulses. It is a general concern in the study of structural intermediates by X-ray crystallography that rearrangements induced by the radiation damage may dominate over biologically relevant structural changes. This is especially true when only a low fraction of protein molecules are in one particular activated state or when early intermediate states with small conformational changes are under investigation. For a functional interpretation of time-resolved structural data, it is therefore vital to minimize the radiation damage as much as possible.

In previous studies of bR, it has been shown that X-ray doses as low as 0.06 MGy lead to structural alterations^42^. At doses ∼1.2 MGy, far below the suggested maximal dose of 30 MGy for Cryo^53^,^54^, several X-ray-induced structural changes appear near the retinal-binding site. Since the spectroscopic properties of bR are strongly coupled to the isomerization of retinal and structural rearrangements of the surrounding amino acids and water molecules, X-ray exposure alone can lead to the formation of a bR species with active-like spectroscopic and structural characteristics^41^,^55^.

Femtosecond X-ray pulses at XFELs have been demonstrated both theoretically^3^ and experimentally^24^ to efficiently outrun the radiation damage processes with the exception of highly radiation sensitive metal clusters^27^. In agreement with this, our bR SFX structure shows no signs of radiation damage in the Asp and Glu residues, where we observed depletion of electron density in our earlier Cryo-structure obtained by conventional crystallography (Supplementary Fig. 3). Intriguingly, the electron density for carboxylic residues is not always better defined when using data from the room temperature measurements SMX and SFX, as compared with the measurements under cryo-conditions. For example, for residue Glu166 the cryo-data gives a very well-resolved electron density for the side chain at 1.5 *σ* contour level, while the room temperature SMX and SFX structures at this contour level are depleted of density for this residue. In this case, even the protein backbone trace differs between these structures and indicates a mobile region of the protein. The structure obtained at cryogenic temperatures thus seems to favour one conformational state yielding well-defined electron density, albeit a slightly different conformation than for the two room temperature structures. Similar effects of remodelling of protein conformational distributions upon crystal cryocooling were analysed in 30 different proteins by Fraser *et al*.^56^. Moreover, the common effect of unit cell shrinkage at cryogenic temperatures^57^ observed also in this case can be associated to a change around Glu166. At cryogenic temperature, the N-terminal turn of the helix F changes its conformation and extends Glu166 backbone by 3.9 Å (ca. 5 Å for side chain) towards the next molecule forming crystal contact by double H bonding to hydroxyl and amide of Ser132 (Supplementary Fig. 2). This shift is also accompanied by the formation of additional crystal contact between hydroxyl of Ser169 and carbonyl of Gly72. Similarly, the protein C terminus at cryogenic temperature shows one additional residue resolved in electron density, Glu232, which forms crystals contact to Lys129. These new interactions approximately parallel to the long c axis of crystallographic unit cell may be contributing to the shrinkage of the *c* axis by ∼10 Å. We also observed a small but significant difference within the unconstrained region of the retinal β-ionone ring (Fig. 2). These changes can probably be attributed to conformational motions that are obscured at cryogenic temperatures. In a broader context, cryo-conditions may interfere with certain ligand conformations present at physiological temperatures. If the energy barrier between different conformational states is small, cooling down the crystals may preferentially favour one of them in the crystal structure. This mechanism may yield better-defined electron density maps, but at the expense of losing information about the unconstrained dynamics of the protein and bound ligand, obscuring information that could be useful in context of a structure-based drug design or simulation approaches. Moreover, numerical sorting techniques applied to the very large serial crystallography data sets may resolve multiple ligand conformations not accessible from a single cryo-cooled X-ray structure.

The native-like lipidic environment of LCP is ideal not only for crystallization of membrane proteins, but also as a favourable medium for the investigation of structural dynamics. The difference Fourier maps presented here are a demonstration of how time-resolved pump-probe experiments with the LCP injector can be used to investigate structural changes, using TR-SFX at an XFEL in a sample efficient fashion. Overall with a hit rate of 5–10% and collection at 30 Hz, the data in our study can be collected with only ∼1 mg of protein comparing very favourably to previous time-resolved studies at atomic resolution with protein consumption in the hundreds of milligrams.

A complication, which both kinetic spectroscopy and crystallography have to overcome, is that at any given time point after activation only a fraction of the protein is in one particular conformation. We chose the 1 ms time point for our TR-SFX experiments, since the M intermediate is a key component of the photocycle and can be highly enriched^58^. However, we used a laser with femtosecond pulses for excitation, which reduced the amount of activated bR to ∼13% (compare Supplementary Fig. 4). In future experiments the use of a femtosecond laser will allow us to probe ultrafast structural changes of the retinal ligand, but with the tradeoff of a lower portion of photoactivated protein compared with using a laser with longer pulse length. This is because the lifetime of the initial excited state of retinal is in the order of a few hundred femtoseconds^59^ and only partially converts into activated bR^47^. A more extensive characterization of the bR photocycle, is thus best divided into two parts. First, the photochemical reaction of the retinal chromophore with a femtosecond laser that allows the probing of ultrafast reactions, but leads to lower levels of activation. Second, the characterization of the slower protein dynamics in the nanosecond to millisecond range using a laser with longer pulse length to maximize activation levels. Especially in the latter time regime, it will become feasible to determine a series of difference electron density maps, each needing less than a milligram of protein, and follow the accumulation of structural intermediates, while they evolve over time. Time-resolved absorption spectroscopy will provide further guidance to determining the time points of maximal enrichment of single intermediates within the crystal lattice. In comparison to other methods such as freeze trapping of intermediates, the TR-SFX method provides real time resolution and allows collection of data without the radiation damage. In the near future, the combination of a sample efficient LCP injector and TR-SFX as demonstrated here thus provides the methodological basis for condensing the kinetics of bR activation into a single movie to illustrate the molecular details of retinal protein activation.

## Methods

### Sample preparation

bR (UniProtKB P02945) purification and crystallization was conducted under dim red light or in the dark^30^. The protein was solubilized with 1.7% β-octyl glucoside (Anagrade) detergent from purple membranes in 50 mM NaH_2_PO_4_ pH 6.9 (GERBU Biotechnik GmbH), the soluble fraction purified by size exclusion chromatography (TSK G3000SW gel filtration column, TOSOH Bioscience, equilibrated with 1.2% β-OG in 25 mM NaH_2_PO_4_ pH 5.5) and concentrated to 15 mg ml^−1^ (Ultracel-50 k). To obtain the sufficient quantity of LCP with crystals, the crystallization was performed in Hamilton gas-tight syringes^60^. The set-up consisted of two 100 μl Hamilton syringes connected by a coupler. Initially, one syringe contained pre-mixed monoolein based LCP with bR and the other precipitant (29–38% (w/v) polyethylene glycol 2,000 (Fluka Analytical) and 100 mM Sorensen phosphate buffer pH 5.6 (KH2PO4 and Na2HPO4 from GERBU). Roughly 20 μl of the LCP was extruded into another syringe to form a LCP tube surrounded by crystallization solution. Within 3 days a high density of microcrystals formed within the LCP.

Shortly before the experiment, the precipitant was removed and a small amount of monoolein was added to obtain homogeneous LCP with suspension of crystals. To avoid LCP phase transition, while injecting into the vacuum of the experimental chamber, we supplemented the sample with 10% MAG 7.9, which has a lower-phase transition temperature than monoolein. Before loading into the LCP injector, the crystals were light adapted for ∼1 min using intense microscope light with a yellow >515 nm long-pass filter. This step was included to convert bR into the light-adapted dark state with full occupancy of all-*trans* retinal^61^. Immediately after the crystals were loaded into the LCP injector reservoir via a syringe adaptor.

### Pump-probe experiment

The TR-SFX pump-probe experiment was performed at the Coherent X-ray Imaging experimental station^62^ with integrated optical pump-probe laser set-up^63^. During our experiment, the XFEL pulses were constrained by a parallel experiment to a pulse length of 75 fs and an energy of 6.74 keV, which limited the highest possible resolution to 2.3 Å with the available detector geometry. X-ray transmission was set to 2% to avoid Bragg peak saturation on the Cornell-SLAC Pixel array detector detector.

The LCP stream from the injector was aligned with the X-ray beam and an optical pump laser (Ti:sapphire oscillator, 50 fs pulse length, 20 μJ energy (530 nm wavelength) synchronized to the XFEL. X-ray pulses during measurements were delivered at 120 Hz repetition rate with the laser pulses at a rate of 30 Hz. This experimental set-up resulted in a cycle of four images, the first of that had a 1 ms time delay between excitation with the optical laser and the probing XFEL pulse. To clear away the LCP from the 80 μm focal spot of the pump laser before the next cycle of four frames is collected, the bR microcrystals were dispersed at a flow speed of 4.22 μm ms^−1^.

The collected data was preprocessed with Cheetah^64^ to select images containing diffraction patterns. These images were further sorted according to an event code introduced during data collection that marked them as exposed and not exposed by the optical pump laser.

After sorting, images were indexed and integrated using CrystFEL 0.6.0+06b84ba3 modified with an improved multiprocessing framework for indexing. The dark data set was merged using CrystFEL version 0.6.1, and the 1 ms data set was merged using CrystFEL 0.6.0+732d177f. The indexing ambiguity in space group P6_3_ was resolved using ambigator in CrystFEL. The data sets were merged using partialator in CrystFEL, with three cycles of scaling without partiality modelling, merging reflections up to 0.3 nm^−1^ higher than a conservative resolution limit estimated for each individual crystal, rejecting saturated reflections (those containing any pixel ∼14,000 detector units) and rejecting crystals where the relative *B* factor was >100 A^2^ or >−100 A^2^.

### Model building and refinement

For structural refinement (bRdark) a data set consisting of every fourth image from the pump-probe cycle was merged with a data set collected without pump laser (dark_bR). The resolution limit of the diffraction signal in the merged intensities was judged as 2.3 Å, based on signal-to-noise ratios, CC*, visual inspection of the density and suggestions by the PDB Redo web server^65^, that also corresponds to the edge of the detector. To minimize bias, the structure of bR was solved in Phaser^66^ with Sensory rhodopsin II (PDB: 1H68^33^) as a molecular replacement model. Then the structure was rebuilt automatically using phenix.autobuild^67^ with simulated annealing refinement steps, followed by manual building in Coot^68^ and refinement with Refmac5^69^. One translation-libration-screw (TLS) group was used for the whole-protein chain.

Fourier difference maps were calculated between the dark_bR data set and every first image from the pump-probe cycle corresponding to a 1 ms time delay. Difference Fourier maps where calculated using Phenix tool phenix.fobs_minus_fobs_map. The same unit cell dimensions of *a*=*b*=62.0 Å and *c*=110 Å were used for molecular replacement, refinement and electron density maps calculations for all data sets. The details of data collection and refinement are presented in Table 1.

Dark–state-subtracted electron density maps were calculated as follows: the bR1ms and bR data without laser (dark_bR) were scaled with Scaleit (including Wilson scaling) and fast Fourier transform (FFT) of ccp4 was used to calculate dark–state-subtracted electron density maps n*F*obs(bR1ms)−m*F*obs(dark_bR) maps with *n* kept at 1 and varying *m* as specified in the left column of the Supplementary Fig. 4. To evaluate the fraction of bR1ms, we tested different values of *m* and compared the corresponding electron density maps with known M state structure (Supplementary Fig. 4). This approach is similar to the calculations of extrapolated difference density maps in ref. 49 and the approach described in ref. 70 except that the map is not extrapolated to 100% M state and even so the M state features can be well distinguished. Simultaneous occupancy refinement of dark and M state was performed in the following procedure: the chain B of pdb 1CWQ corresponding to the M state was merged into one pdb with our bRdark-state structure. This pdb was subjected to occupancy only refinement in phenix.refine against bR1ms data to obtain an estimation of relative fractions of dark and M state.

### Time-resolved visible absorption spectroscopy

The time-resolved absorption measurements were performed in the pump-probe configuration. Microsecond pulses from a Xe-flash lamp (L7684, Hamamatsu Photonics) were split into two, to provide the probe (95%) and reference (5%) pulses. The reference pulse was used to correct the pulse-to-pulse fluctuation of the probe light intensity. The 4-ns, 532-nm second harmonic generation (SHG) output of a Nd-YAG laser (Minilite-II, Continum) was used as the pump pulse to excite the photocycle reaction of bR. The pump and probe pulses were focused onto the sample with the beam diameter of 250 and 100 μm, respectively. The pump energy was reduced to 0.2 μJ to avoid photodegradation of the sample.

The delay time between the pump and probe pulses was adjusted by delay generators (DG645 and DG535, Stanford Research Systems) with <40 ns jitter. The repetition rates of pump and probe were 2 and 20 Hz, respectively. The probe and reference intensities were measured using fiber-coupled spectrometers (USB2000+, Ocean Optics), controlled by LabVIEW (National Instruments). Twenty-two different time delays were measured and 40 measurements were accumulated at each delay time. The curve fitting of the time trace data was performed with three exponentials by the Igor software (WaveMetrics). The bR sample was packed between the quartz windows with a spacer of 50 μm thickness and kept at 293 K.

### Data availability

Coordinates and structure factors have been deposited in the Protein Data Bank under accession code 5J7A. The following link https://figshare.com/s/9fab6bf5fe8bc8e3d7b9 contains files for an interactive PyMOL session to examine the dark–state-subtracted electron density map^48^. All other data associated with this manuscript are available from the authors on reasonable request.

## Additional information

**How to cite this article:** Nogly, P. *et al*. Lipidic cubic phase injector is a viable crystal delivery system for time-resolved serial crystallography. *Nat. Commun.* 7:12314 doi: 10.1038/ncomms12314 (2016).

## Supplementary Material

Supplementary Information Supplementary Figures 1-5.

Supplementary Movie 1 The movie illustrates enhancement of the M state features in the electron density when increasing fractions (0 to 0.87) of the dark state are subtracted from the bR1ms data (nFobs(bR1ms)-mFobs(dark_bR)). Bacteriorhodopsin is shown in dark state (purple C atoms, PDB code: 5J7A) and M state (pink C atoms, PDB code: 1DZE). The electron density was drawn in Pymol at 1 RMSD, except the part depicting reorganization of water 402, which was drawn at 0.5 RMSD. The movie shows a sequence of images corresponding to Supplementary Fig. 4.

## Figures and Tables

**Table 1:** Data collection and refinement statistics.

	**bR**_**dark**_ **(5J7A; this work)**	**bR**_**1 ms**_ **(this work)**	**SMX (4X31)**	**Cryo (4X32)**
*Data collection*				
X-ray source	CXI, LCLS	CXI, LCLS	ID13, ESRF	PXI-X06SA, SLS
Detector	CSPAD	CSPAD	Rayonix MX-170 CCD	PILATUS 6 M
Temperature (K)	294	294	294	100
Wavelength (Å)	1.83	1.83	0.954	1.000
Beam size (μm)	1.5 × 1.5	1.5 × 1.5	2 × 3	50 × 10
Average crystal size (μm)	3–15 × 3–15 × 1–3	3–15 × 3–15 × 1–3	5–40 × 5–40 × 1–5	50 × 50 × 10
Pulse duration/no. of photons	75 fs (nominal)/6.57e10	75 fs (nominal)/6.57e10	25 ms	150 ms per image
Space group	*P*6_3_	*P*6_3_	*P*6_3_	*P*6_3_
Unit cell parameters (Å)	*a*=*b*=62.62 *c*=111.3	*a*=*b*=62.71 *c*=111.2	*a*=*b*=62.8, *c*=109.7	*a*=*b*=60.5, *c*=101.5
Indexed images	13,804	7,820	5,691	2,532
Crystals merged	13,132	7,117	5,691	1
Total/unique reflections	1,799,769/11027	714,736/10,981	1,223,766/9,655	234,541/16,643
Resolution range (Å)	21.9−2.30 (2.41−2.30)	21.9−2.30 (2.41−2.30)	36.56−2.40 (2.46−2.40)	46.57−1.90 (1.94−1.90)
Completeness (%)	99.98 (99.86)	99.36 (92.83)	100.0 (100.0)	100.0 (100.0)
Multiplicity	163 (16.8)	65.1 (4.9)	127 (88.8)	14.1 (14.3)
〈*I*/*σ(I)*〉	6.56 (1.58)	4.88 (1.56)	3.57 (1.16)	17.90 (1.80)
CC*	0.998 (0.862)	0.998 (0.761)	0.981 (0.658)	1.000 (0.841)
*R*_split_† (%) or *R*_p.i.m._ (cryo; %)	11.5 (66.3)	9.52 (87.4)	22.4 (107)	2.6 (50)
Matthews coefficient° *V*_M_ (Å^3^ Da^−1^)	2.44	2.44	2.50	2.21
Solvent content (%)	49.65	49.65	50.76	44.27
*B* factor Wilson plot (Å^2^)	31.3	72.0	45.2	33.4
				
*Refinement*				
Resolution range (Å)	21.66−2.3 (2.36−2.30)		31.40−2.40 (2.46−2.40)	52.42−1.90 (1.95−1.90)
No. of reflections (total/test set)	10,660/650		9,192/441	15,773/841
*R*_work_/*R*_free_ (%)	19.0/22.0		20.5/24.9	17.1/21.4
				
No. of atoms				
Overall	1,805		1,848	1,877
Protein	1,711		1,756	1,723
Retinal	20		20	20
Water	17		10	30
Lipids and other	57		62	104
				
Average *B* factors (Å^2^)				
Overall	19.19		40.47	28.50
Protein	17.88		39.04	27.11
Retinal	16.13		52.67	24.61
Water	43.31		55.94	36.52
Lipids and other	52.39		74.47	49.93
				
r.m.s.d.'s				
Bond lengths (Å)	0.008		0.008	0.009
Bond angles (°)	1.15		1.01	1.21
Ramachandran favoured (%)	99.1		98.2	98.9
Ramachandran outliers (%)	0.0		0.4	0.0

bR, bacteriorhodopsin; CSPAD, Cornell-SLAC Pixel array detector; CXI, Coherent X-ray Imaging; LCLS, Linac Coherent Light Source; SMX, serial millisecond crystallography; SLS, Swiss Light Source.

Values for the SMX and Cryo-structures are taken from ref. 30 and shown for easier comparison.

Error estimates for SFX unit cell para metres are the s.d.'s of the observed distributions of parameters.

CC^*^=[2CC_1/2_/(1+CC_1/2_)]^1/2^.

^†^*R*_split_=(1/2^1/2^)Σ_*hkl*_|*I*_even_−*I*_odd_|/1/2Σ_*hkl*_|*I*_even_+*I*_odd_|.
