# Supplementary material for: Lipidic cubic phase injector is a viable crystal delivery system for time-resolved serial crystallography
Source: Nat Commun. 2016 Aug 22;7:12314. doi: 10.1038/ncomms12314 (PMC4996941; doi:10.1038/ncomms12314)
Supplement: Supplementary Information — Supplementary Figures 1-5. [file ncomms12314-s1.pdf]

Supplementary Information

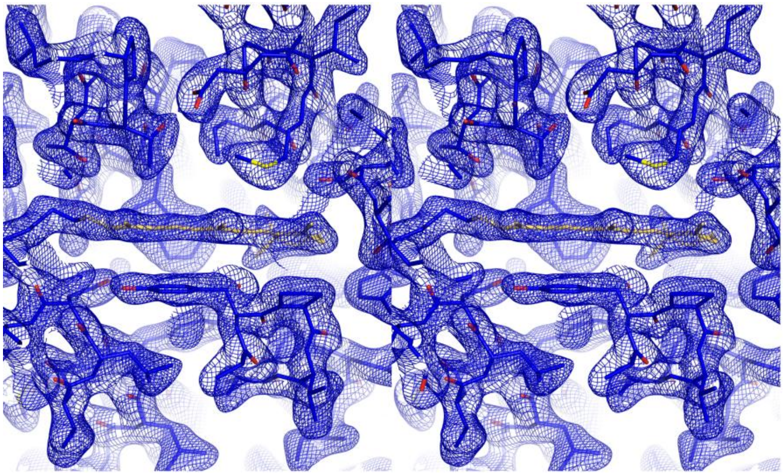

Supplementary Figure 1 | Stereo view on retinal site for bRdark SFX structure. 2*F*o-*F*c map is drawn at 1 σ.

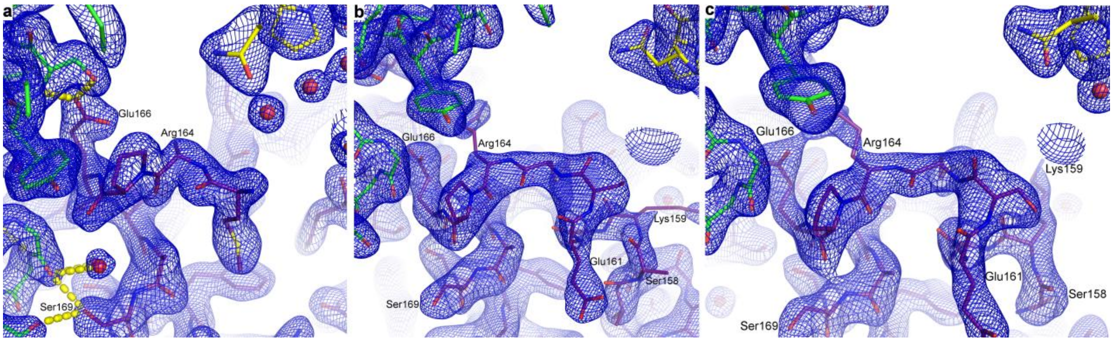

Supplementary Figure 2 | View on the EF loop (magenta C atoms) of dark state bacteriorhodopsin and the neighbor molecules (green and yellow C atoms) in Cryo (a), SMX (b) and SFX (c) datasets. 2*F*o-*F*c map is drawn at 1 σ and yellow dashed lines indicate intermolecular polar contacts as identified in PyMOL (up to 3.2 Å). In the Cryo structure the EF loop exhibits a different conformation which is accompanied by presence of additional intermolecular hydrogen bonds as compared to the two room temperature structures SMX and SFX. The EF loop (residues 163-167) in all three structures have well defined conformation in the electron density. However, the upstream residues at the end of helix E (residues 155-162) have either weaker density in some parts (SMX) or backbone gaps (Cryo, SFX) indicating some flexibility of that part of structure independently from the temperature of measurement.

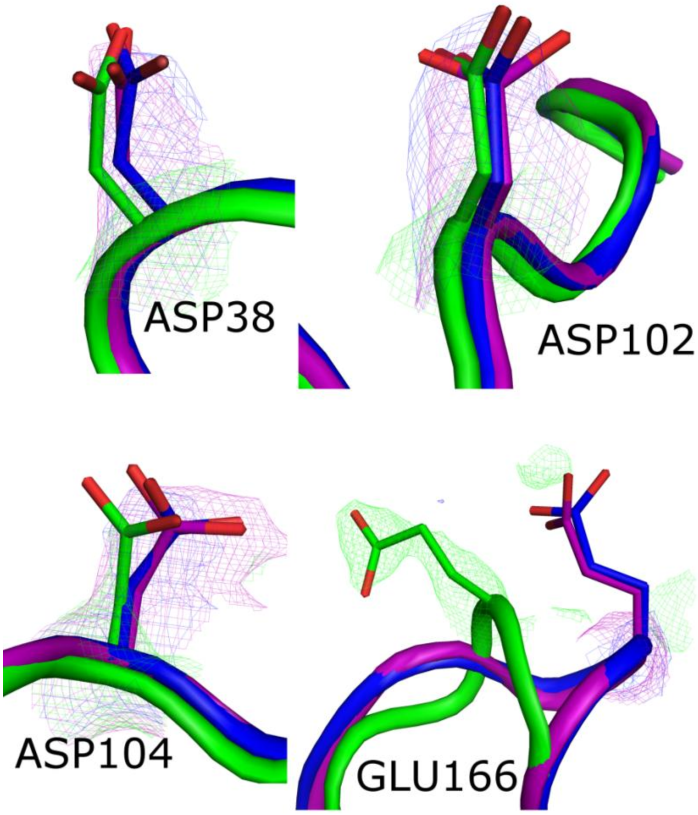

Supplementary Figure 3 | Electron density ($2F_o$-F_c, 1.5σ) for exemplary carboxylate residues in the dark state bacteriorhodopsin SFX (blue), SMX (Purple) and Cryo (green) structures. In many cases observations compatible with early signs of radiation damage can be made in case of the data collected by conventional cryo-crystallography but not in the room temperature data collected by serial crystallography. However, in case of Glu166 cryo data collection resulted in clearly better density. Crystal shrinkage coinciding with cryo treatment results in a different crystal packing where the Glu166 side chain is stabilized via contacts to a symmetry related molecule in the cryo dataset, leading to better defined density. The lack of electron density in such residues is thus unlikely to be due to radiation damage but rather reflects the loop flexibility preserved at room temperature.

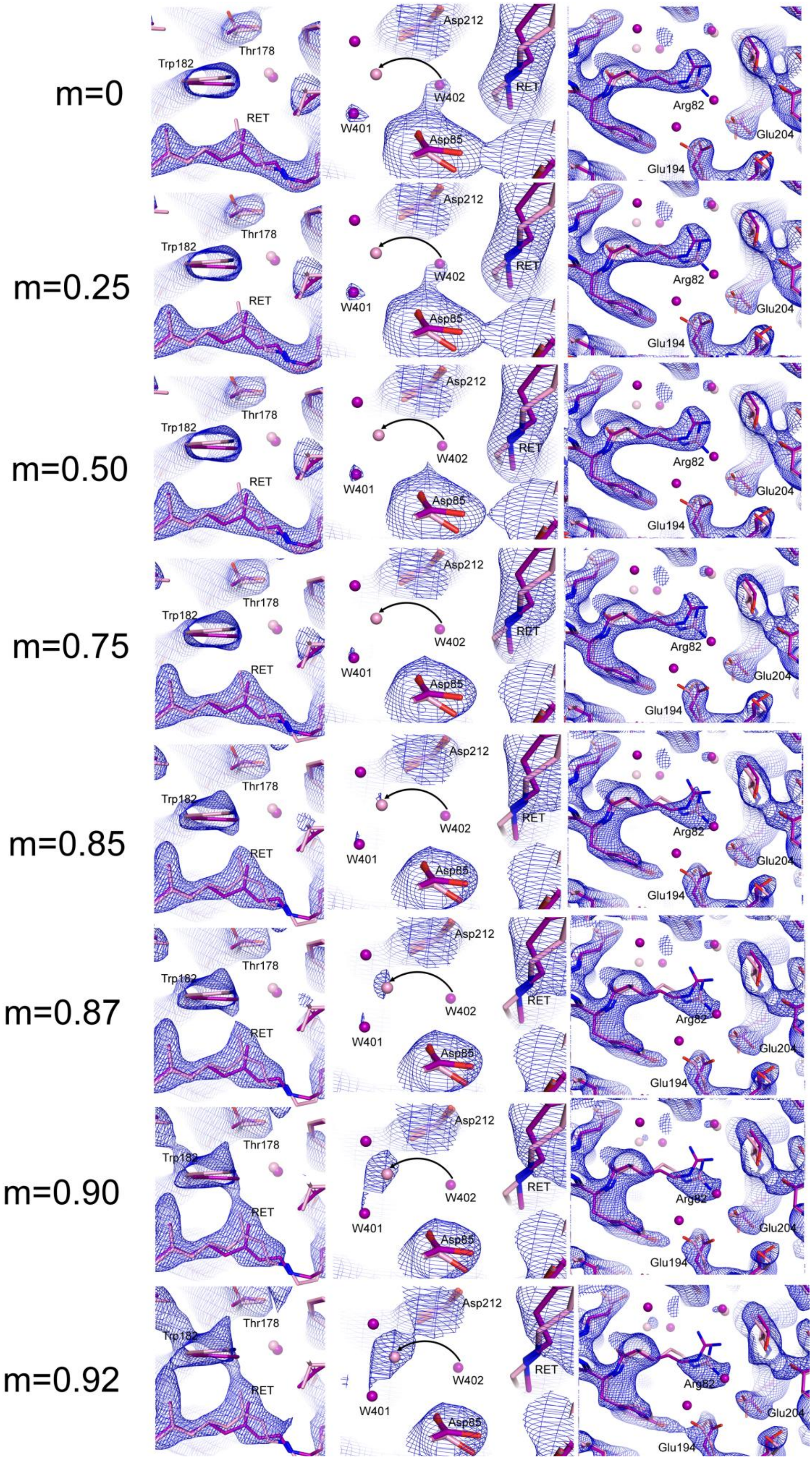
m=0
m=0.25
m=0.50
m=0.75
m=0.85
m=0.87
m=0.90
m=0.92
Trp182
Thr178
RET
Asp212
W402
W401
Asp85
Arg82
Glu204
Glu194

Supplementary Figure 4 | Gradual subtraction of dark state contribution in the bR_{1ms} data. The bR_{1ms} and bR data without laser were scaled with Scaleit (including Wilson scaling) and FFT of ccp4 was used to calculate n*F*obs(bR_{1ms})-**m***F*obs(dark_bR) maps with n kept at 1 and varying **m** as specified in the left column of the figure. The electron density was drawn in Pymol at 1 RMSD, except the middle panel, which was drawn at 0.5 RMSD to depict reorganization of water 402. BR is shown in dark state (purple C atoms) and M state (pink C atoms). The obtained dark state subtracted electron density maps seem to correspond slightly better to the M state structure deposited with PDB code 1dze than the 1cwq:chainB. Overall the features corresponding to the M state (PDB code: 1dze) are well highlighted at an m value of 0.87 indicating an M state contribution (1-**m**) of about 13%. This is in good agreement with occupancy refinement yielding 11-13% occupancy for the M state (depending on the starting parameters). Supplementary Movie 1 shows the above images in a sequence and link https://figshare.com/s/9fab6bf5fe8bc8e3d7b9 contains files for comparison in PyMOL session of maps for m=0 and m=87.

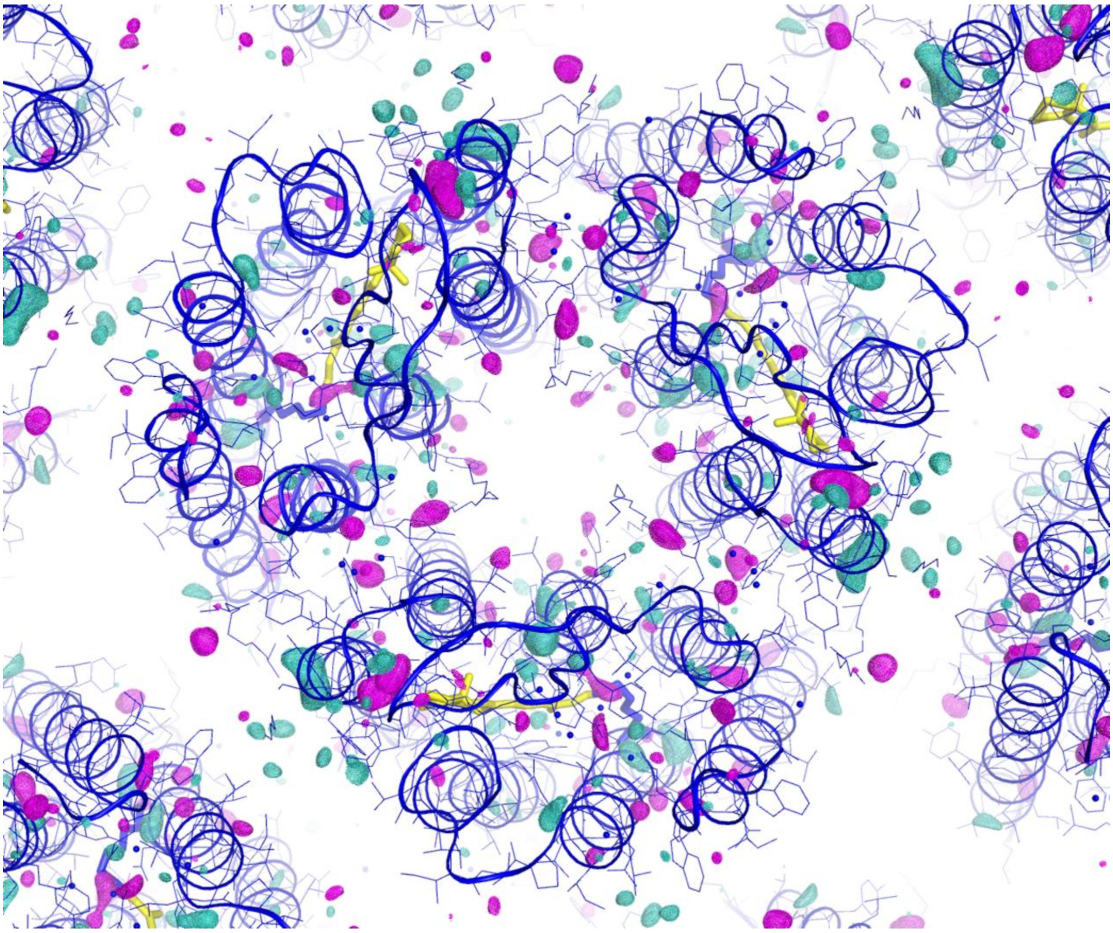

Supplementary Figure 5 | SFX structure of a bacteriorhodopsin trimer. The Fourier difference map *F*obs(bR1ms)-*F*obs(dark_bR) is presented around the central trimer at 3 σ.
